# Simple organic structure directing agents for synthesizing nanocrystalline zeolites

**DOI:** 10.1039/c7sc02858j

**Published:** 2017-10-05

**Authors:** Eva M. Gallego, Cecilia Paris, M. Rocío Díaz-Rey, Marta E. Martínez-Armero, Joaquín Martínez-Triguero, Cristina Martínez, Manuel Moliner, Avelino Corma

**Affiliations:** a Instituto de Tecnología Química , Universitat Politècnica de València-Consejo Superior de Investigaciones Científicas , Avenida de los Naranjos s/n , 46022 València , Spain . Email: acorma@itq.upv.es ; Email: mmoliner@itq.upv.es

## Abstract

The synthesis of the ZSM-5 and beta zeolites in their nanosized form has been achieved by using simple alkyl-substituted mono-cationic cyclic ammonium cations as OSDA molecules. The particular combination of a cyclic fragment and a short linear alkyl-chain group (preferentially C_4_) within the monocationic OSDA molecules allows directing the crystallization of nanosized zeolites with excellent solid yields (above 90%). Interestingly, the formation of the nanosized ZSM-5 and beta zeolites mostly depends on the size and nature of the cyclic fragment of the OSDA molecule, resulting in all cases in nanocrystalline solids with homogeneous distributions of particle sizes (∼10–25 nm) and controlled Si/Al molar ratios (∼15–30). The achieved nanosized ZSM-5 and beta zeolites have been extensively characterized by different techniques to study their physico-chemical properties, such as chemical composition, pore accessibility or Brønsted acidity, among others. Moreover, the catalytic properties of the nanosized ZSM-5 and beta zeolites have been evaluated for different chemical reactions, including methanol-to-olefins (MTO) in the case of ZSM-5, and alkylation of benzene with propylene to obtain cumene and oligomerization of light olefins to liquid fuels in the case of beta, observing in all cases improved catalytic activity and product selectivity towards target products when compared to related catalysts.

## Abstract 2

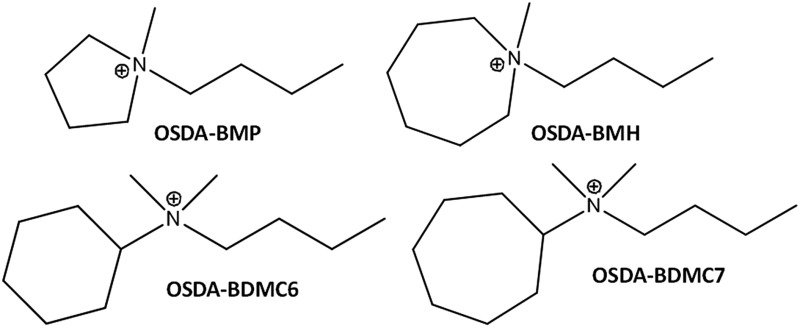
The synthesis of ZSM-5 and beta zeolites in their nanosized form has been achieved by using simple alkyl-substituted mono-cationic cyclic ammonium cations as OSDA molecules.

## 1. Introduction

Zeolites are microporous crystalline materials with pores and cavities with uniform size and shape in the molecular range (∼3–15 Å), which can be prepared with different pore topologies, chemical compositions, and/or crystal sizes.^1^,^2^ This large versatility has allowed their industrial implementation in diverse applications, including gas adsorption and separation, oil conversion, petrochemistry and for the preparation of chemicals and fine chemicals.^3^–^5^ More recently, there has also been a significant scientific and commercial interest in the synthesis of zeolites for environmental purposes.^6^

Some of the zeolite properties can be tuned during their preparation. Among them, the ability of controlling their crystal sizes is one of the most determinant factors.^7^,^8^ Indeed, the particle size can notoriously influence the catalytic activity and the product selectivity of a specific chemical process, particularly when bulky reactants/products are involved, or when different consecutive reactions take place and the presence of large diffusion paths could result in the formation of undesired products.^9^ In the last years, many efforts of the scientific community have been devoted to the efficient preparation of zeolites with crystal sizes in the nanometer scale (below 100 nm), while also controlling the chemical composition of the achieved crystalline materials and the resultant solid yields.^10^

Probably the two zeolites most studied in their nanosized forms are ZSM-5 and beta, including synthesis procedures and catalytic applications.^11^–^15^ This fact can be explained because these two zeolite materials are extensively employed as industrial catalysts,^16^–^18^ and their preparation as nanocrystals could remarkably improve their catalytic capacities for many chemical processes.

For instance, the synthesis of the nanosized beta zeolite, with sizes ranging from 10 to 100 nm, was first described in the 90's using tetraethylammonium (TEA) as organic structure directing agent (OSDA)^11^ but, unfortunately, this synthesis methodology resulted in low synthesis solid yields (below 50%) when the Si/Al molar ratio was increased to values above 10.^11^,^12^ In the case of the ZSM-5 zeolite, similar trends are found in the literature when using tetrapropylammonium (TPA) cation as OSDA, but only a few optimized synthesis procedures report the preparation of high-silica nano-ZSM-5 material in the presence of TPA with acceptable solid yields (above 80%) and crystal sizes below 100 nm, ranging from 70 to 100 nm.^14^

In general, the improvement of the synthesis solid yield and the decrease of the particle size for beta and ZSM-5 zeolites, respectively, have been mainly achieved by employing large and bulky organic molecules as OSDAs.^19^–^23^ On the one hand, high-silica beta zeolites (Si/Al ∼10–30) with small particle sizes (∼50–100 nm) and increased solid yields (above 80%) have been achieved using 4,4′-trimethylenebis(*N*-methyl,*N*-benzyl-piperidinium),^19^ 3,10-diazoniabicyclo[10.2.2]hexadeca-12,14,15-triene-3,3,10,10-tetramethyl-dichloride,^20^ or polydiallyldimethylammonium chloride.^21^ On the other hand, the synthesis of ZSM-5 zeolites as nanosheet crystals (thickness below 5–10 nm) has been promoted by the use of bifunctional dicationic molecules containing a long aliphatic chain (C_22_H_45_–N^+^(CH_3_)_2_–C_6_H_12_–N^+^(CH_3_)_2_–C_6_H_13_).^22^,^23^ In this particular case, the diammonium fraction allows the formation of the ZSM-5 layers, while the long hydrophobic chain prevents the zeolite growth along the normal direction of the sheet by the formation of a micellar structure.^22^

Despite the fact that these recent descriptions using large and bulky OSDA molecules allow controlling, at least partially, the physico-chemical properties of the resultant nanozeolites (crystal size and/or chemical composition), the efficient synthesis of the high-silica nanocrystalline beta and ZSM-5 zeolites with high-solid yields but using more simple and affordable OSDA molecules, is still a challenging and relevant issue. Indeed, the catalysts prepared using sophisticated OSDA molecules implicate high costs associated to the manufacture of the organic compounds, requiring many synthesis/purification steps and/or expensive organic sources, and precluding their potential industrial applicability. In a very illustrative recent perspective, Mintova *et al.* have enumerated the real challenges for the next years in the synthesis of nanosized zeolites and, according to the authors, the most important challenge in the field is the potential implementation of nanozeolites under real industrial applications.^24^ To achieve this, the reduction of the manufacturing costs is a critical issue, and this requires the efficient preparation of nanozeolites using simple OSDAs, or even under OSDA-free conditions.^24^

Very recently, Burton from ExxonMobil and our group have described the synthesis of the nanocrystalline form of ZSM-5 and beta zeolites in a broad range of Si/Al molar ratios and with good synthesis yields (above 85%) using large alkyl-substituted dicationic OSDA molecules.^25^,^26^ The dicationic nature of these molecules combined with the alkyl chains, mainly butyl or pentyl chains, suggests that these OSDAs could act as surfactant-type organic molecules favoring the crystallization of beta or ZSM-5 in their nanocrystalline forms. Those preliminary results are quite interesting, since they open an attractive research pathway to design industrially relevant zeolites in their nanosized form using less-expensive OSDA molecules, which interestingly, would not require the presence of very large aliphatic alkyl chains. Nevertheless, there is still room for optimizing the synthesis procedure of these two industrial zeolites in their nanosized form using simpler organic molecules, and even more important, it would be highly desirable to create general methodologies using simple OSDA molecules for the preparation of other nanosized zeolite structures.

**Fig. 1:**
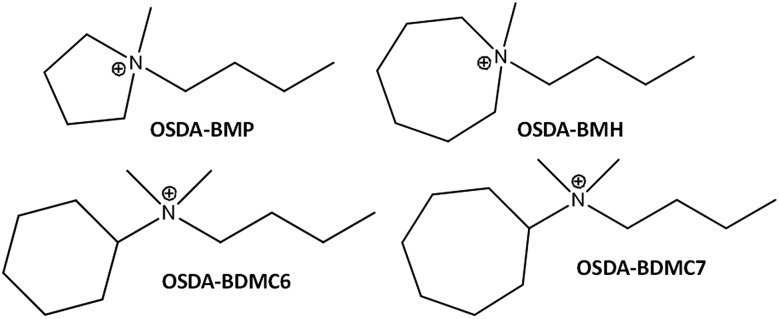
Organic structure directing agents (OSDAs) used for the synthesis of the nanosized beta and ZSM-5 zeolites.

Herein, we will show the efficient preparation of the nanosized forms of ZSM-5 and beta zeolites with controlled Si/Al molar ratios (∼15–30), high solid yields (above 90%), and very homogeneous crystal sizes (∼10–25 nm), using simple alkyl-substituted mono-cationic cyclic ammonium cations as OSDA molecules. These dual-function monocationic OSDA-molecules are quaternary ammonium cations combining a cyclic group and a short linear alkyl-chain group (preferentially C_4_, see Fig. 1) and, depending on the size and nature of the cyclic fragment (cyclic ammonium cations or cycloalkyl-substituted ammonium cations), the crystallization of ZSM-5 and beta zeolites can be controlled. The nanosized ZSM-5 and beta zeolites have been tested as acid catalysts in diverse industrially relevant reactions, such as methanol-to-olefins (MTO) in the case of ZSM-5, and alkylation of benzene with propylene to obtain cumene and oligomerization of light olefins to liquid fuels in the case of beta. In all cases, the synthesized nanozeolites clearly show a better catalytic performance, including both, catalyst lifetime and product selectivities towards the desired products, as compared to other related zeolites.

These monocationic dual-function-based OSDA molecules that combine a cyclic fragment and a butyl alkyl group have been rarely explored in the literature for zeolite synthesis, and we believe that the results presented here could pave the way for a general and efficient synthesis approach to direct the crystallization of other nanosized zeolites by properly selecting the cyclic/alkyl groups.

## 2. Experimental section

### 2.1. Synthesis of the OSDAs

#### OSDA-BMP: *N*-butyl-*N*-methylpyrrolidinium

In a two-neck round flask equipped with stirring and a glass condenser, 15 g of 1-butylpyrrolidine (0.118 moles) was dissolved in 200 ml of chloroform. The solution was cooled down in an ice bath (0 °C) and left around 30 minutes under stirring. Then, 33.47 g of iodomethane (0.236 moles) was added dropwise for 1 hour. When the mixture reached 25 °C, the resulting solution was reacted for 72 h. Once the reaction was finished, the solvent was removed under reduced pressure and the product was crystalized in a cooled ethanol-ethyl acetate solution. Finally, crystallized *N*-butyl-*N*-methylpyrrolidinium iodide (OSDA-BMP) was recovered by filtration, obtaining 27.6 g (0.1025 moles) of the final product. The resultant iodide salt was exchanged to the hydroxide form using a commercially available hydroxide ion exchange resin (Dowex SBR).

##### Elemental analysis (C_9_H_20_IN)

%N: 5.203; %C: 40.073; %H: 7.460 (exp.). %N: 5.20; %C: 40.16; %H: 7.49; %I: 47.15 (calc.).

^1^H-NMR (300 MHz, CDCl_3_), *δ* (ppm): 0.96 (*t*, CH_3_, 3H); 1.43 (m, CH_2_, 2H); 1.75 (m, CH_2_, 2H); 2.28 (m, CH_2_; 4H); 3.24 (s, CH_3_, 3H); 3.61 (m, CH_2_, 2H); 3.77 (m, CH_2_, 4H).

^13^C-NMR (75 MHz, CDCl_3_), *δ* (ppm): 13.76 (CH_3_); 19.67 (CH_2_); 21.73 (CH_2_, 2); 25.96 (CH_2_); 49.30 (CH_3_); 64.23 (CH_2_); 64.80 (CH_2_, 2).

#### OSDA-BMH: *N*-butyl-*N*-methylhexamethyleneiminium

OSDA-BMH, was synthetized through a modification of the synthetic protocol described by Pomelli *et al.*^27^

##### Step 1: synthesis of *N*-butylhexamethyleneiminium bromide

The reaction was carried under argon atmosphere. 60.88 g of 1-bromobutane (0.445 moles) was added during 1 hour, dropwise under stirring, to a dry-DMF hexamethyleneimine solution (44.14 g; 0.4436 moles, 250 ml). Then, the resulting mixture was heated up to 70 °C and reacted overnight. A white crystalline solid was formed under the course of the reaction. When the reaction was concluded, the crude was cooled in an ice-bath to complete crystallization of the solid. The product was then separated by filtration and the crystals were washed with an ethyl acetate–hexane solution to remove traces of dimethylformamide. Finally, the product was dried at reduced pressure.

###### Elemental analysis (C_10_H_22_BrN)

%N: 5.969; %C: 50.717; %H: 9.903 (exp.). %N: 5.93; %C: 50.85; %H: 9.39; %Br: 33.83 (calc.).

##### Step 2: synthesis of *N*-butylhexamethyleneamine

The bromide salt obtained in the previous step (50.39 g; 0.2133 moles) was dissolved in 400 ml of distilled water. Then, 22.61 g of anhydrous Na_2_CO_3_ (0.2133 moles) was added and the solution was left to react under strong stirring at room temperature over 1 hour. The free amine was formed in the upper part of the biphasic solution. Then, the solution was decanted and the organic phase was reserved. The organic phase was washed with NaCl saturated solution (100 ml) and dried with anhydrous MgSO_4_. Finally, 1-*N*-butylhexamethyleneimine was isolated as a dense transparent liquid after filtration.

^1^H-NMR (300 MHz, CDCl_3_), *δ* (ppm): 0.90 (*t*, CH_3_, 3H); 1.30 (m, CH_2_, 2H); 1.44 (m, CH_2_, 2H); 1.62–1.65 (m, CH_2_; 8H); 2.4 (m, CH_2_, 2H); 2.61 (m, CH_2_, 4H).

^13^C-NMR (75 MHz, CDCl_3_), *δ* (ppm): 14.06 (CH_3_); 20.79 (CH_2_); 26.99 (CH_2_, 2); 27.97 (CH_2_); 29.76 (CH_2_); 55.57 (CH_2_, 2); 58.11 (CH_2_).

##### Step 3: synthesis of *N*-butyl-*N*-methylhexamethyleneiminium

Caution: highly exothermic reaction. A solution of 1-*N*-butylhexametilenimine (21.84 g; 0.141 moles) in 200 ml of chloroform was added to a two-neck round flask, provided with stirring and a glass condenser. The mixture was cooled in an ice-bath (0 °C) and then, 39.91 g of iodomethane was added dropwise (0.281 moles) over 1 hour time. When the solution was stabilized at room temperature, the mixture was left to react for 72 h. Once the reaction was finished, the solvent was removed under reduced pressure and the product was isolated after crystallization with ethyl acetate. *N*-Butyl-*N*-methylhexamethyleniminium iodide was achieved as a white solid (40.01 g; 0.1346 moles). The resultant iodide salt was exchanged to the hydroxide form using a commercially available hydroxide ion exchange resin (Dowex SBR).

###### Elemental analysis (C_11_H_24_IN)

%N: 4.654; %C: 44.386; %H: 8.489 (exp.). %N: 4.71; %C: 44.45; %H: 8.14; %I: 42.70 (calc.).

^1^H-NMR (300 MHz, D_2_O), *δ* (ppm): 0.89 (*t*, CH_3_, 3H); 1.32 (m, CH_2_, 2H); 1.63–1.70 (m, CH_2_; 6H); 1.82 (m, CH_2_; 2H); 2.94 (s, CH_3_, 3H); 3.23 (m, CH_2_, 2H); 3.27–3.44 (m, CH_2_, 4H).

^13^C-NMR (75 MHz, D_2_O), *δ* (ppm): 12.90 (CH_3_); 19.28 (CH_2_); 21.40 (CH_2_, 2); 23.94 (CH_2_); 27.24 (CH_2_, 2); 50.17 (CH_3_); 64.38 (CH_2_, 2); 65.01 (CH_2_).

#### OSDA-BDMC6: *N*-butyl-*N*,*N*-dimethylcyclohexylammonium

Five equivalents of 1-bromobutane (44.05 g, 0.3215 moles) were added to a solution of *N*,*N*-dimethylcyclohexylamine (8.18 g, 0.0643 moles) in acetonitrile (100 ml) and, then, the resulting solution was heated to reflux (aprox. 90 °C) for 48 h. The solution was cooled to room temperature and the solvent evaporated under reduced pressure. A white crystalline precipitate was formed. Finally, the solid was dissolved in a small volume of chloroform and recrystallized by adding a cold ethyl acetate–diethyl ether solution. The resultant bromide salt was exchanged to the hydroxide form using a commercially available hydroxide ion exchange resin (Dowex SBR).

##### Elemental analysis (C_12_H_26_BrN)

%N: 5.389; %C: 54.426; %H: 10.882 (exp.). %N: 5.30; %C: 54.54; %H: 9.92; %Br: 30.24 (calc.).

^1^H-NMR (300 MHz, CDCl_3_, *δ* (ppm)): 0.90 (*t*, CH_3_, 3H); 1.09 (m, CH_2_, 1H); 1.38 (m, CH_2_; 6H); 1.64 (m, CH_2_, 3H); 1.87 (m, CH_2_; 2H); 2.10 (m, CH_2_, 2H); 3.16 (s, CH_3_, 6H); 3.42 (m, CH_2_, 2H); 3.45 (m, CH, 1H).

^13^C-NMR (75 MHz, CDCl_3_), *δ* (ppm): 13.67 (CH_3_); 19.67 (CH_2_); 24.46 (CH_2_); 24.60 (CH_2_); 25.22 (CH_2_, 2); 26.28 (CH_2_, 2), 48.73 (CH_3_, 2); 62.53 (CH_2_); 71.91 (CH).

#### OSDA-BDMC7: *N*-butyl-*N*,*N*-dimethylcycloheptylammonium

##### Step 1: synthesis of *N*,*N*-dimethylcycloheptylamine

11.80 g (0.104 moles) of cycloheptylamine was introduced into a two-neck round flask provided with stirring and a glass condenser. The amine was cooled in an ice-bath (0 °C) and, under vigorous stirring, 6 equivalents of formaldehyde (18.79 g, 0.626 moles) were added followed by 6 equivalents of formic acid (28.80 g, 0.626 moles). Once the mixture was stabilized at room temperature, the solution was heated at 80 °C overnight. Then, the solution was cooled to room temperature and a solution of NaOH 8 M was added dropwise until a basic pH was reached (pH = 13).

Then, the solution was decanted and extracted with chloroform (3 × 30 ml). The organic phase was reserved and dried with anhydrous MgSO_4_. After filtration, the solvent was evaporated under reduced pressure, and the *N*,*N*-dimethylcycloheptylamine was isolated as a dense light yellow liquid.

^1^H-NMR (300 MHz, CDCl_3_, *δ* (ppm)): 1.42 (m, CH_2_; 5H); 1.51 (m, CH_2_, 4H); 1.68 (m, CH_2_, 3H); 1.77 (m, CH_2_, 2H); 2.22 (s, CH_3_; 6H); 2.48 (m, CH_2_, 1H).

^13^C-NMR (75 MHz, CDCl_3_), *δ* (ppm): 25.55 (CH_2_, 2); 28.14 (CH_2_, 2); 29.38 (CH_2_, 2); 40.65 (CH_3_, 2); 64.94 (CH).

##### Step-2: *N*-butyl-*N*,*N*-dimethylcycloheptanammonium

The *N*,*N*-dimethylcycloheptylamine (14.36 g, 0.1017 moles) was dissolved in 200 ml of acetonitrile. Then, 5 equivalents of 1-bromobutane (41.82 g, 0.3052 moles) were added and the mixture was heated at reflux overnight. When the reaction was finished, the solvent was removed under reduced pressure and a white residue was formed. Finally, the residue was dissolved in a small volume of chloroform and the *N*-butyl-*N*,*N*-dimethylcycloheptanammonium bromide was crystallized by addition of a ethyl acetate–hexane solution. The resultant bromide salt was exchanged to the hydroxide form using a commercially available hydroxide ion exchange resin (Dowex SBR).

###### Elemental analysis (C_13_H_28_BrN)

%N: 5.212; %C: 55.808; %H: 10.265 (exp.). %N: 5.03; %C: 56.11; %H: 10.14; %Br: 28.71 (calc.).

^1^H-NMR (300 MHz, CDCl_3_, *δ* (ppm)): 0.96 (*t*, CH_3_, 3H); 1.38 (m, CH_2_, 2H); 1.52 (m, CH_2_; 6H); 1.73 (m, CH_2_, 6H); 2.15 (m, CH_2_; 2H); 3.24 (s, CH_3_, 6H); 3.52 (m, CH_2_, 2H); 3.61 (m, CH, 1H).

^13^C-NMR (75 MHz, CDCl_3_), *δ* (ppm): 13.70 (CH_3_); 19.71 (CH_2_); 24.64 (CH_2_); 24.79 (CH_2_, 2H); 27.13 (CH_2_, 2); 27.37 (CH_2_, 2), 48.81 (CH_3_, 2); 62.64 (CH_2_); 73.56 (CH).

### 2.2. Synthesis of zeolites

For the synthesis of zeolites, the following procedure was employed: the required content of the aqueous solutions of the OSDAs in their hydroxide form was mixed with the required content of the silicon source (Ludox AS-40, Sigma-Aldrich, 40 wt%) and aluminum source [Al(OH)_3_, Sigma-Aldrich, 58%]. The resulting synthesis mixture was maintained under stirring for the required time to evaporate the excess of water until achieving the desired gel concentration. The compositions of the synthesis gels were: SiO_2_: 0.017–0.033 Al_2_O_3_: 0.4 OSDA: 15H_2_O, where OSDA can be BMP, BMH, BDMC6 or BDMC7.

The resultant gels were charged into stainless steel autoclaves with Teflon liners, and the crystallizations were conducted at 150 °C for 14 days under static conditions. The solids were filtered, washed with water, and dried at 100 °C. Calcination of the solid samples to remove the organic molecules occluded within the crystals was performed in air at 580 °C for 6 h.

### 2.3. Characterization

Powder X-ray diffraction (PXRD) measurements were performed with a multisample Philips X'Pert diffractometer equipped with a graphite monochromator, operating at 40 kV and 35 mA, and using Cu Kα radiation (*λ* = 0.1542 nm). The chemical analyses were carried out in a Varian 715-ES ICP-Optical Emission spectrometer, after solid dissolution in HNO_3_/HCl/HF aqueous solution.

The morphology of the samples was studied by field emission scanning electron microscopy (FESEM) using a ZEISS Ultra-55 microscope and by field emission transmission electron microscopy (TEM) using a JEM 2100F microscope.

Textural properties were obtained from the N_2_ adsorption–desorption isotherms measured at 77 K with a Micromeritics ASAP 2020 apparatus.

Solid NMR spectra were recorded at room temperature with a Bruker AV 400 MAS spectrometer. ^27^Al MAS NMR spectra were recorded at 104.2 MHz with a spinning rate of 10 kHz and 9° pulse length of 0.5 μs with a 1 s repetition time. ^27^Al chemical shift was referred to Al^3+^(H_2_O)_6_.

Infrared spectra were measured with a Nicolet 710 FT IR spectrometer. Pyridine adsorption–desorption experiments were made on self-supported wafers (10 mg cm^–1^) of original samples previously activated at 673 K and 10^–2^ Pa for 2 hours. After wafer activation, the base spectrum was recorded and pyridine vapor (6.5 × 10^2^ Pa) was admitted in the vacuum IR cell and adsorbed onto the zeolite. Desorption of pyridine was performed under vacuum over three consecutive one-hour periods of heating at 423, 523 and 623 K, each of them followed by the IR measurement at room temperature. The spectra were scaled according to the sample weight.

### 2.4. Catalytic test

#### Methanol to olefins (MTO)

The catalyst was pelletized, crushed and sieved into a particle size range of 0.25–0.42 mm. 50 mg of sample was mixed with 2 g quartz (Fluka) before being introduced into the fixed-bed reactor (7 mm diameter). N_2_ (20 ml min^–1^) was bubbled in methanol hold at 23 °C, giving a WHSV = 10 h^–1^. The catalyst was first activated with an air flow of 80 ml min^–1^ for 1 h at 540 °C, and then the temperature was decreased to reaction conditions (450 °C). Each experiment was analyzed every 5 minutes with an online gas chromatograph (Bruker 450GC, with PONA and Al_2_O_3_-plot capillary columns, and two FID detectors). Conversion and selectivities were considered on carbon basis.

#### Liquid phase alkylation of benzene with propylene

Liquid phase alkylation of benzene with propylene was carried out with the acid zeolites, pelletized, crushed, and sieved at 0.25–0.42 mm diameter. 0.2 g of the pelletized samples were diluted with SiC (>0.64 mm) to obtain a bed volume of 3.6 cm^3^. The reaction was performed in an automated high-pressure stainless steel fixed-bed reactor, at 3.5 MPa, 125 °C, WHSV = 25 h^–1^ with a benzene to propylene (B/P) molar ratio of 3.5. Before reaction, the catalysts were activated *in situ* at 200 °C in N_2_ flow (100 ml min^–1^). Then, the reactor was cooled to the reaction temperature in a flow of N_2_ (100 ml min^–1^). The two reactants were fed to the reactor as liquids by means of two electronic liquid flow controllers, and the pressure was maintained during the reaction by means of a back pressure regulator. The composition of the outlet stream was analyzed online on a Varian-450 gas chromatograph equipped with a 30 m 5% phenyl–95% dimethylpolysiloxante capillary column connected to a flame ionization detector (FID). More details can be found in a previous study.^28^

#### Oligomerization of 1-pentene

The catalytic oligomerization experiments were performed in an automated high-pressure stainless steel fixed-bed reactor, at 4.0 MPa, 200 °C, and WHSV = 14.3 and 25 h^–1^. Under these conditions, the reaction occurs in the liquid phase. The zeolites were pelletized, crushed and sieved to recover the particles with sizes in the 0.25–0.42 mm fraction. 0.13 g of the pelletized samples were diluted with SiC (0.64–0.82 mm) to obtain a bed volume of 4.0 cm^3^. Before reaction, the catalysts were activated *in situ* by increasing temperature to 520 °C in N_2_ flow (200 ml min^–1^) at a rate of 2.0 °C min^–1^ and further calcining in air flow (200 ml min^–1^) at 520 °C for 5 h. Then, the reactor was cooled to the reaction temperature in a flow of N_2_ (200 ml min^–1^). The olefinic mixture was fed to the reactor as liquid by means of a Gilson piston pump, and the pressure was controlled during the reaction by means of a back pressure regulator. 1-Pentene was co-fed with *n*-heptane in a 60 : 40 1-pentene/*n*-heptane molar ratio. The full reactor outlet stream was vaporized and analyzed online with a Varian 3800 gas chromatograph, equipped with a 25 m, 0.25 mm × 1.2 μm CP-Sil 5 CB column and a FID detector. Finally, the C_5_^+^ mixture was condensed for further analysis by simulated distillation, excluding *n*-heptane from the naphtha fraction. For discussion, the selectivity results are referred to as the naphtha, diesel, and heavy product fractions, determined by simulated distillation according to the following cut points:

Naphtha: C_5_–173.9 °C.

Diesel: 173.9–391.1 °C.

Heavy fraction: 391.1–1000 °C.

## 3. Results and discussion

### 3.1. Zeolite synthesis and characterization

Different monocationic cyclic organic molecules have been initially studied as OSDAs to investigate the effect of the ring size and the nature of the ammonium-based molecule, including cyclic ammonium and cycloalkyl-substituted ammonium cations (see Fig. 1). As seen in Fig. 1, the ring size of the cyclic ammonium cations has been varied from pyrrolidinium (*N*-butyl-*N*-methylpyrrolidinium, BMP) to azepanium (*N*-butyl-*N*-methylhexamethyleneiminium, BMH) and, in addition, two different sized cycloalkyl-substituted ammonium cations have also been proposed, *N*-butyl-*N*,*N*-dimethylcyclohexylammonium (BDMC6) and *N*-butyl-*N*,*N*-dimethylcycloheptylammonium (BDMC7). These organic molecules have been tested as OSDAs for the zeolite synthesis under the following conditions: SiO_2_: 0.017–0.033 Al_2_O_3_: 0.4 OSDA: 15H_2_O, where OSDA can be BMP, BMH, BDMC6 or BDMC7, and the crystallization was conducted at 150 °C for 14 days under static conditions.

#### Synthesis and characterization of nanosized ZSM-5

**Fig. 2:**
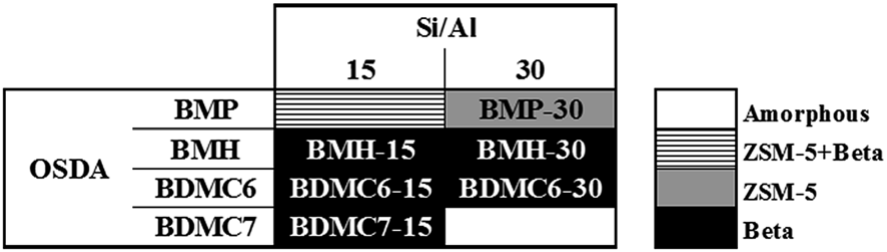
Phases achieved using the OSDAs shown in Fig. 1 (synthesis conditions: OSDA/Si = 0.4; H_2_O/Si = 15; *T* = 150 °C; *t* = 14 d).

**Fig. 3:**
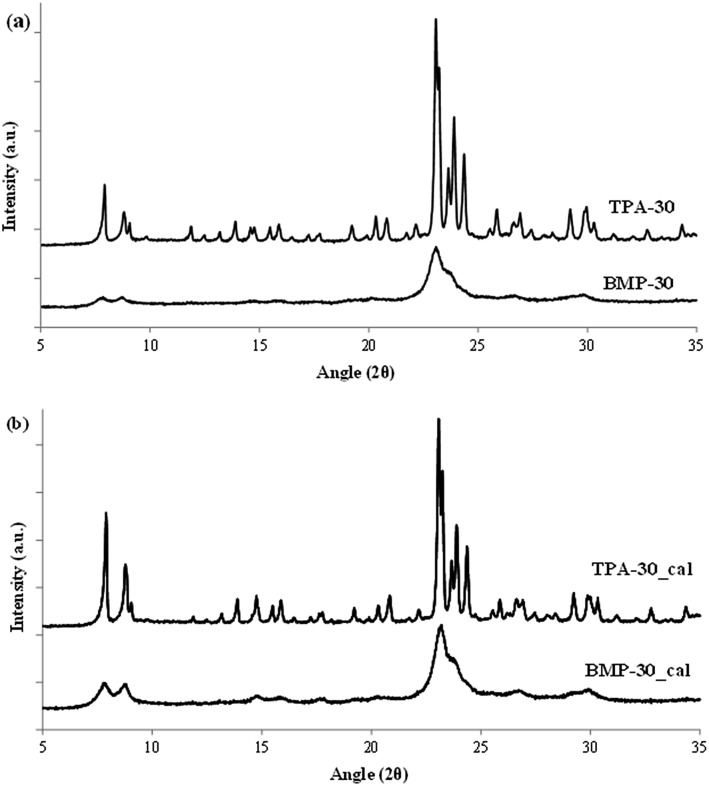
PXRD patterns of the as-prepared (a) and calcined (b) ZSM-5 zeolites.

As seen in Fig. 2, the OSDA-BMP cyclic ammonium cation, which contains the smallest heterocycle (pyrrolidinium) shows the preferential crystallization of the ZSM-5 zeolite. The PXRD pattern of the achieved solid when the Si/Al molar ratio was fixed at 30, shows the presence of very low-intense and broad peaks, which suggest the crystallization of this material as very small crystallites (see BMP-30 in Fig. 3a). It is important to remark that the solid yield achieved after the hydrothermal synthesis is above 90 wt%, based on the inorganic sources introduced in the initial gel mixture. In fact, the chemical analysis of the BMP-30 sample gives a final Si/Al molar ratio of 33.2, which is analogous to the initial Si/Al molar ratio in the gel, confirming the excellent incorporation of the different heteroatoms present in the synthesis mixture into the final crystalline solid.

**Fig. 4:**
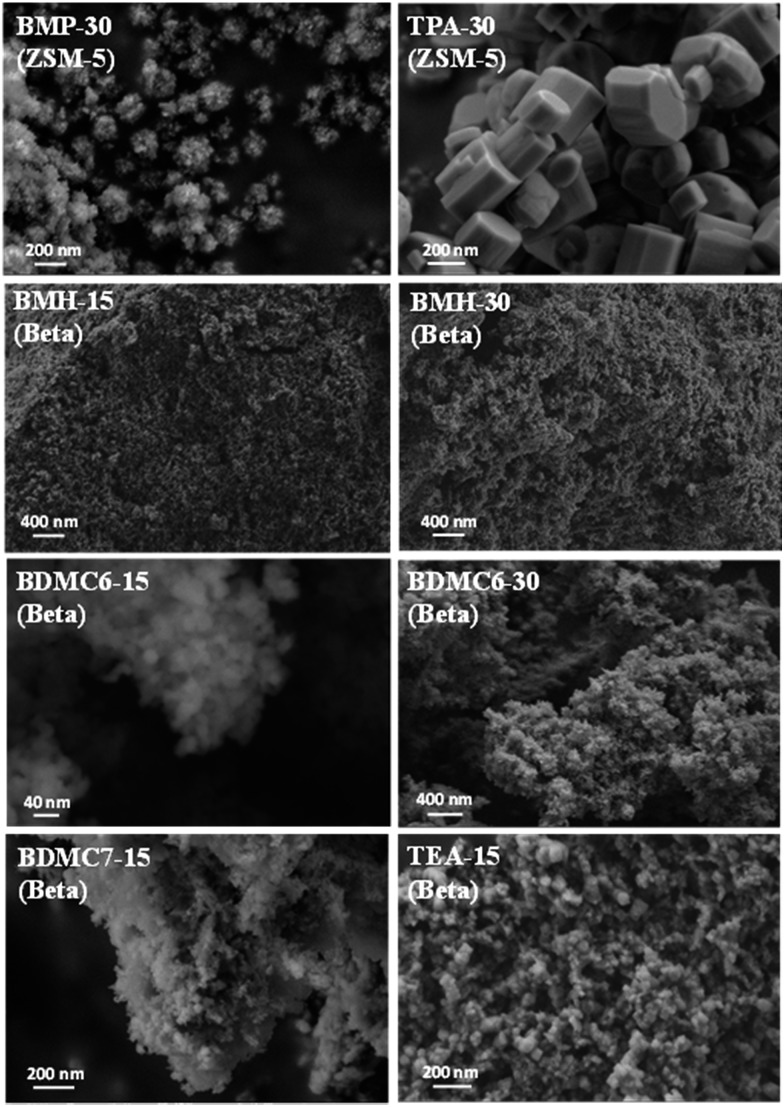
FE-SEM images of the prepared ZSM-5 and beta zeolites.

**Fig. 5:**
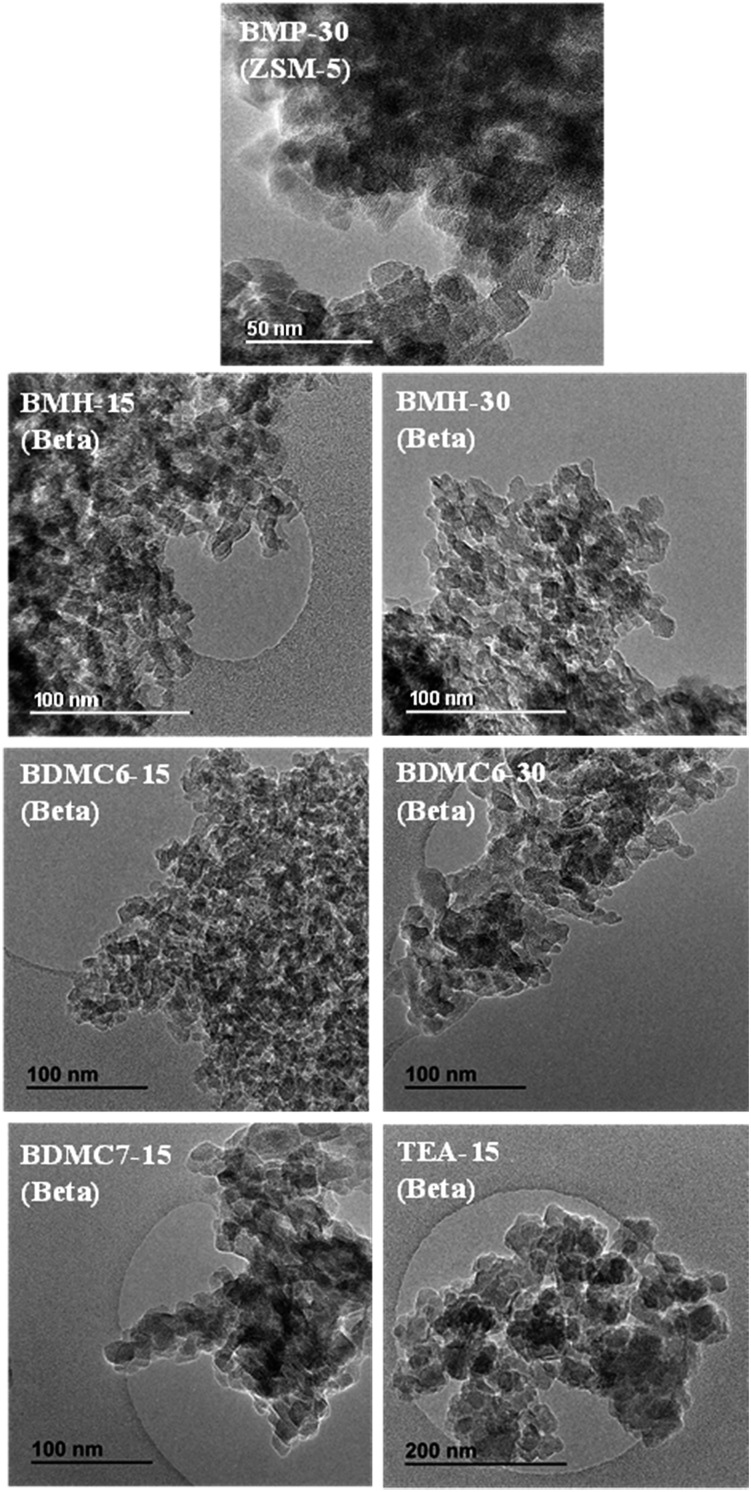
TEM images of the prepared ZSM-5 and beta zeolites.

The homogeneous crystallization of the BMP-30 sample and its nanocrystalline nature has been studied by scanning and transmission electron microscopy (SEM and TEM). SEM images of the BMP-30 sample indicate the homogeneous distribution of very small crystallites within the entire sample (see BMP-30 in Fig. 4), whereas TEM images allow measuring an average crystal size of ∼15 nm (see BMP-30 in Fig. 5a).

At this point, and for comparison purposes, the synthesis of ZSM-5 was carried out under identical conditions to those employed for the preparation of the BMP-30 sample, but using the preferred OSDA employed in the literature to synthesize the ZSM-5, *i.e.* tetrapropylammonium (TPA).^29^ When using TPA under these particular conditions, it can be seen that the ZSM-5 zeolite is also achieved, but its PXRD pattern suggests the formation of remarkable bigger crystal sizes compared to BMP-30 (see TPA-30 in Fig. 3a). The SEM image of the TPA-30 sample confirms the presence of larger crystals, with an average size of 200 nm (see TPA-30 in Fig. 4). In addition, it has been noticed that the solid yield obtained after the zeolite crystallization is also lower than the one achieved above using BMP as OSDA (less than 70%). These results highlight the benefits of using a butyl-substituted pyrrolidium-based molecule as OSDA for the efficient synthesis of ZSM-5 with very small crystallites (∼15 nm).

Both ZSM-5 samples with similar Si/Al molar ratios (∼30, see BMP-30 and TPA-30 in Table 1), have been calcined in air at 580 °C to remove the occluded OSDA molecules. The textural properties of these calcined ZSM-5 samples have been measured by N_2_ adsorption, observing a much higher BET surface area and external surface area for the nanosized BMP-30 sample (514 and 194 m^2^ g^–1^, respectively, see Table 1) compared to TPA-30 (360 and 11 m^2^ g^–1^, respectively, see Table 1), as it could be predicted from the significant lower crystal size of the former.

**Table 1:** Chemical analyses of the as-prepared nanosized beta zeolites^*a*^

Sample	(Si/Al)_ICP_	Yield (wt%)	Crystal size (nm)	Area BET	Ext. surface area	Microp. area
BMP-30	33.2	92	10–15	514.2	193.8	320.4
TPA-30	31.8	68	200–250	360.2	11.1	349.1
BMH-15	15.6	98	10–15	732.7	333.4	399.3
BMH-30	29.9	91	10–15	681.8	252.2	429.6
BDMC6-15	15.6	95	10–15	686.9	303.3	378.8
BDMC6-30	26.2	96	15–20	N.M.	N.M.	N.M.
BDMC7-15	16.9	98	15–20	N.M.	N.M.	N.M.
TEA-15	15.5	53	35–40	530.6	134.0	396.7

^*a*^N.M.: not-measured.

**Fig. 6:**
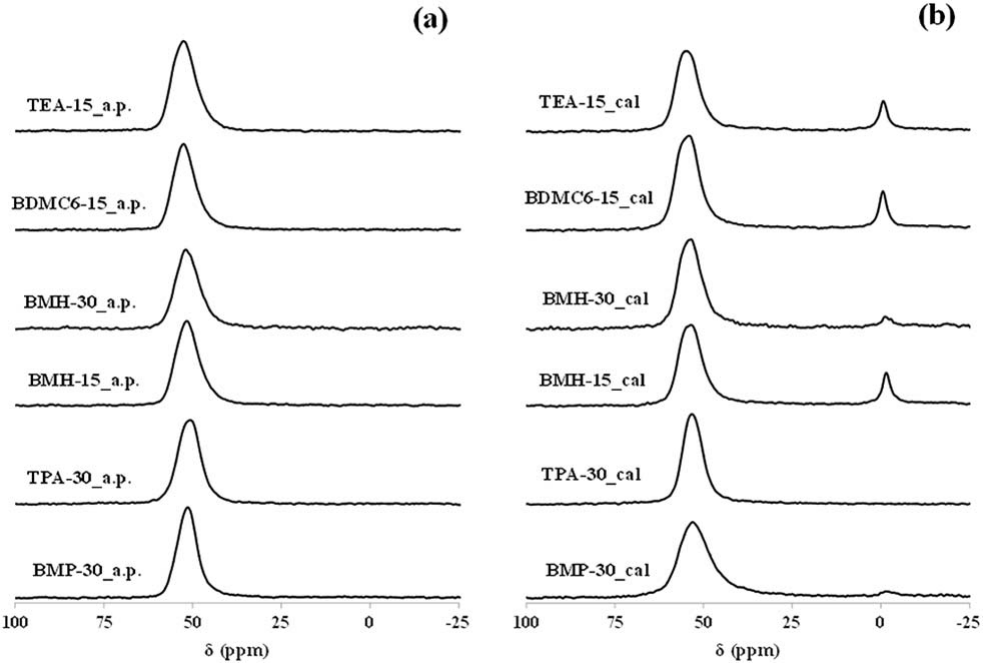
^27^Al MAS NMR spectra of the as-prepared (a) and calcined (b) ZSM-5 and beta zeolites.

The coordination of the aluminum species in the calcined samples has been characterized by ^27^Al MAS NMR spectroscopy. As seen in Fig. 6b, the calcined BMP-30 and TPA-30 samples show the main presence of a signal centered at ∼55 ppm in their corresponding ^27^Al MAS NMR spectra, indicating that practically the entire aluminum species remain in tetrahedral coordination after the calcination treatment. The acid properties of these materials have been evaluated by the adsorption of pyridine as probe molecule, and its posterior desorption at different temperatures (150, 250 and 350 °C), using IR spectroscopy to control and quantify the pyridine adsorption/desorption. The amount of Brønsted and Lewis acid sites per gram of zeolite can be determined from the IR bands centered at 1545 and 1455 cm^–1^, respectively.^30^ The nanosized BMP-30 sample presents a slightly higher Brønsted acidity, particularly after increasing the pyridine desorption temperatures (see B250 and B350 for BMP-30 and TPA-30 in Table 2). On the other hand, the TPA-30 catalyst show lower Lewis acidity than BMP-30 (see Table 2), as it could be expected by the lower proportion of aluminum species in octahedral coordination within the calcined TPA-30 material (see signal centered at ∼0 ppm in their corresponding ^27^Al MAS NMR spectra in Fig. 6).

**Table 2:** Acidity of the calcined zeolites as determined by FT-IR combined with pyridine adsorption–desorption

Sample	Acidity (μmol pyr per g)					
	B150	B250	B350	L150	L250	L350
BMP-30	349	314	242	171	159	151
TPA-30	311	264	174	75	62	38
BMH-15	274	259	176	190	188	173
BMH-30	176	158	119	144	144	137
BDMC6-15	267	220	147	340	330	307
TEA-15	275	238	162	170	167	153

#### Synthesis and characterization of nanosized beta

**Fig. 7:**
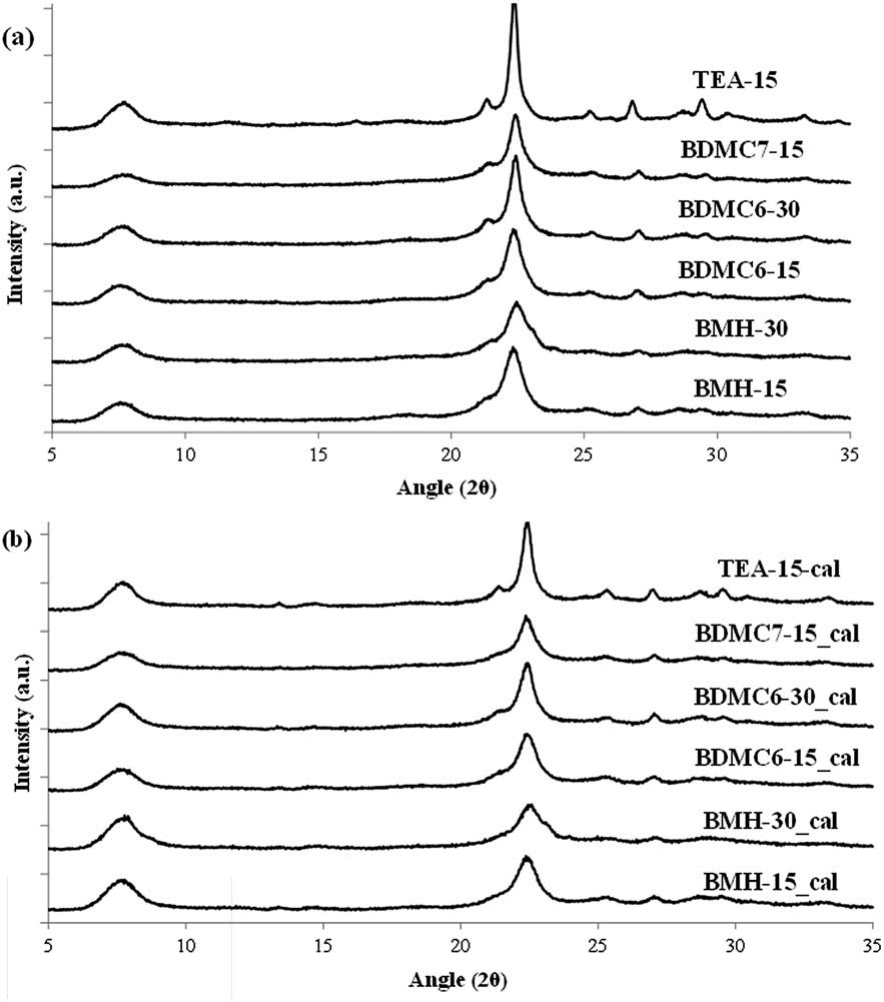
PXRD patterns of the as-prepared (a) and calcined (b) beta zeolites.

When the cyclic ammonium OSDA contains a larger ring size, as the azepanium-based OSDA-BMH (see Fig. 1), the crystallization of beta zeolite is selectively achieved under the synthesis conditions studied (see Fig. 2). These results would suggest that the larger ring size of the azepanium heterocycle would mostly restrict the crystallization of the medium pore ZSM-5 zeolite (10-rings, ∼5–5.5 Å), favoring the nucleation and crystallization of a zeolite presenting larger pores, such as beta zeolite (12-rings, ∼6.5–7 Å). The PXRD patterns of the two solids obtained using the OSDA-BMH reveal the crystallization of the beta zeolite but, as in the case of ZSM-5, they also show the presence of broad diffraction peaks with low intensity (see BMH-15 and BMH-30 in Fig. 7a), which could indicate the formation of small crystallites in both cases. SEM and TEM microscopy confirms the homogeneous formation of very small crystallites for these two beta zeolites, with average particle sizes of 10–15 nm (see BMH-15 and BMH-30 in Fig. 4 and 5). Moreover, and as occurred above when OSDA-BMP was employed for the synthesis of the nanosized ZSM-5 zeolite, the use of OSDA-BMH also resulted in the crystallization of zeolites with excellent solid yields (above 90%, see BMH-15 and BMH-30 in Table 1). The measured chemical compositions of these beta zeolites were 15.6 and 29.9 for BMH-15 and BMH-30, respectively, which were equivalent to the theoretical values fixed for their preparations.

After calcining the above materials in air at 580 °C, their textural properties were measured by N_2_ adsorption, observing in both cases very large BET surface areas (732 and 680 m^2^ g^–1^ for BMH-15 and BMH-30, respectively) and external surface areas (333 and 252 m^2^ g^–1^ for BMH-15 and BMH-30, respectively), as expected from their very low crystal sizes (see Table 1).

In order to demonstrate the benefits of using the OSDA-BMH template, containing the heterocycle ring and the butyl group, we have proposed the synthesis under identical conditions to those employed for the preparation of the BMH-15 zeolite but now using tetraethylammonium (TEA) as OSDA. This molecule has been proposed because it is the preferred OSDA for the synthesis of beta zeolite according to the literature.^31^ As it can be seen in Fig. 7a, the PXRD pattern of the sample synthesized in presence of TEA, TEA-15, shows the crystallization of the beta zeolite as a pure phase. However, the achieved crystalline material shows a very low solid yield (∼50%, see Table 1). It is important to note that similar low solid yields were described in the literature when using TEA as OSDA for the synthesis of nanocrystalline beta zeolite under hydrothermal alkaline conditions.^11^,^12^ The TEA-15 sample shows small crystallites with an average size of ∼35–40 nm (see Table 1 and TEM image in Fig. 5), which nevertheless are larger than those achieved using the OSDA-BMH organic molecule (see Table 1). The larger crystal size observed for the TEA-15 material, results in lower BET and external surface areas (530 and 134 m^2^ g^–1^, respectively, see Table 2) than those values measured for the other nanobeta zeolites with smaller crystallites (see Table 1).

At this point, we would like to study if the OSDA molecules can be extended beyond the alkyl-substituted cyclic ammonium cations to other ammonium-based molecules containing substituted cycloalkyl groups with different ring sizes. In this sense, two new OSDA molecules have been proposed, *N*-butyl-*N*,*N*-dimethylcyclohexylammonium (OSDA-BDMC6) and *N*-butyl-*N*,*N*-dimethylcycloheptylammonium (OSDA-BDMC7). These organic molecules have been tested under the same conditions as those previously described for the cyclic ammonium cations: SiO_2_: 0.017–0.033 Al_2_O_3_: 0.4 OSDA: 15H_2_O, where OSDA can be OSDA-BDMC6 and OSDA-BDMC7, and the crystallization was conducted at 150 °C for 14 days under static conditions.

In this case, the preferred crystallized phase under the selected conditions is beta zeolite (see OSDA-BDMC6 and OSDA-BDMC7 in Fig. 2). The presence of the large cyclohexyl and cycloheptyl groups within the OSDA-BDMC6 and OSDA-BDMC7 organic molecules seem to favor the crystallization of a zeolite with large pores, such as the beta zeolite. Interestingly, the beta zeolites achieved using cycloalkyl-substituted ammonium cations not only show very small crystal sizes (∼15–20 nm), but also these materials have been obtained with excellent solid yields (above 95%, see BDMC6-15, BDMC6-30, and BDMC7-15 in Table 1). These nanocrystalline beta zeolites also show comparable Si/Al molar ratios (∼15–30) to those initially introduced in the synthesis gels (see Table 1), and in addition, the BET and external surface areas of the BDMC6-15 (687 and 303 m^2^ g^–1^, respectively) are higher than the area measured for the nanocrystalline TEA-15 zeolites with larger crystal size (see TEA-15 in Table 1).

The aluminum coordination and the Brønsted acidity of the synthesized nanocrystalline beta zeolites have been analyzed to evaluate their possible use as acid heterogeneous catalysts. All the as-prepared beta zeolites, regardless of the OSDA employed for their preparation or the Si/Al molar ratio, contain all the aluminum species in tetrahedral coordination in their as-prepared forms (see band centered at 55 ppm in the ^27^Al MAS NMR spectra of the BMH-15, BMH-30, BDMC6-15 and TEA-15 samples in Fig. 6a). After calcination in air at 580 °C, the spectra corresponding to the nanocrystalline beta zeolites synthesized with a Si/Al molar ratio of 15, show that a small portion of the aluminum species are extracted from framework positions (see signal at ∼0 ppm in the ^27^Al MAS NMR spectra of the calcined BMH-15, BDMC6-15 and TEA-15 samples in Fig. 6b), but still above 80–85% of the aluminum species remain in tetrahedral coordination (see signal at ∼55 ppm in the ^27^Al MAS NMR spectra of the calcined BMH-15, BDMC6-15 and TEA-15 samples in Fig. 6b). On the other hand, the nanocrystalline beta zeolite synthesized with higher Si/Al molar ratio (∼30, see BMH-30 in Fig. 6b) maintains more than 95% of the aluminum species in tetrahedral coordination after the calcination treatment (see ^27^Al MAS NMR spectrum of the calcined BMH-30 sample in Fig. 6b). Similar results have been obtained in the literature for other nanocrystalline beta zeolites containing comparable Si/Al molar ratios.^26^ Regarding the acid properties of these nanosized beta zeolites, measured by *in situ* FTIR spectroscopy of pyridine adsorption and its posterior desorption at different temperatures, the nanocrystalline beta zeolites synthesized with Si/Al molar ratios of ∼15 present similar Brønsted acidity (see Table 2), while the nanosized beta zeolite synthesized with a Si/Al molar ratio of ∼30 shows the lowest Brønsted acidity, as corresponds to its lower Al content (see BMH-30 in Table 2).

### 3.2. Catalytic results

The nanocrystalline zeolites described above have been tested in different industrially-relevant catalytic reactions and, more specifically, in reactions where, according to the literature, the reduction of the diffusion pathways within zeolite-based catalysts allows improving their catalytic behavior, both in terms of catalyst lifetime and product selectivities towards desired products.^3^ The selected reactions are methanol to olefins (MTO) in the case of ZSM-5, and alkylation of benzene with propylene to obtain cumene and oligomerization of olefins to liquid fuels in the case of beta.

#### Methanol to olefins (MTO)

In the last years, the production of light olefins from methanol has become an important technology, since methanol can be efficiently achieved from non-petroleum sources, such as natural gas, coal or biomass.^32^ Regarding the different MTO catalysts, ZSM-5 is one of the very few zeolites that is now being employed for the methanol to propylene (MTP) process.^33^ The crystal size of the MTO catalysts has been described as a determining factor to improve their catalytic activity, since small crystallites would favor the mass transfer of reactants and products, by shortening the diffusion path lengths, and by lowering the deactivation rates due to inhibition of the excessive coke formation,^34^ while increasing the selectivity to the intermediate propene product.

**Fig. 8:**
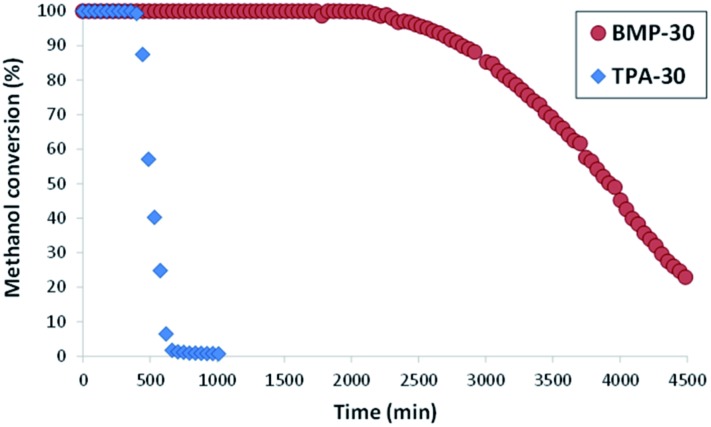
Conversion of methanol using ZSM-5 catalysts (reaction conditions: *T* = 450 °C, WHSV = 10 h^–1^, *W*_cat_ = 50 mg).

**Fig. 9:**
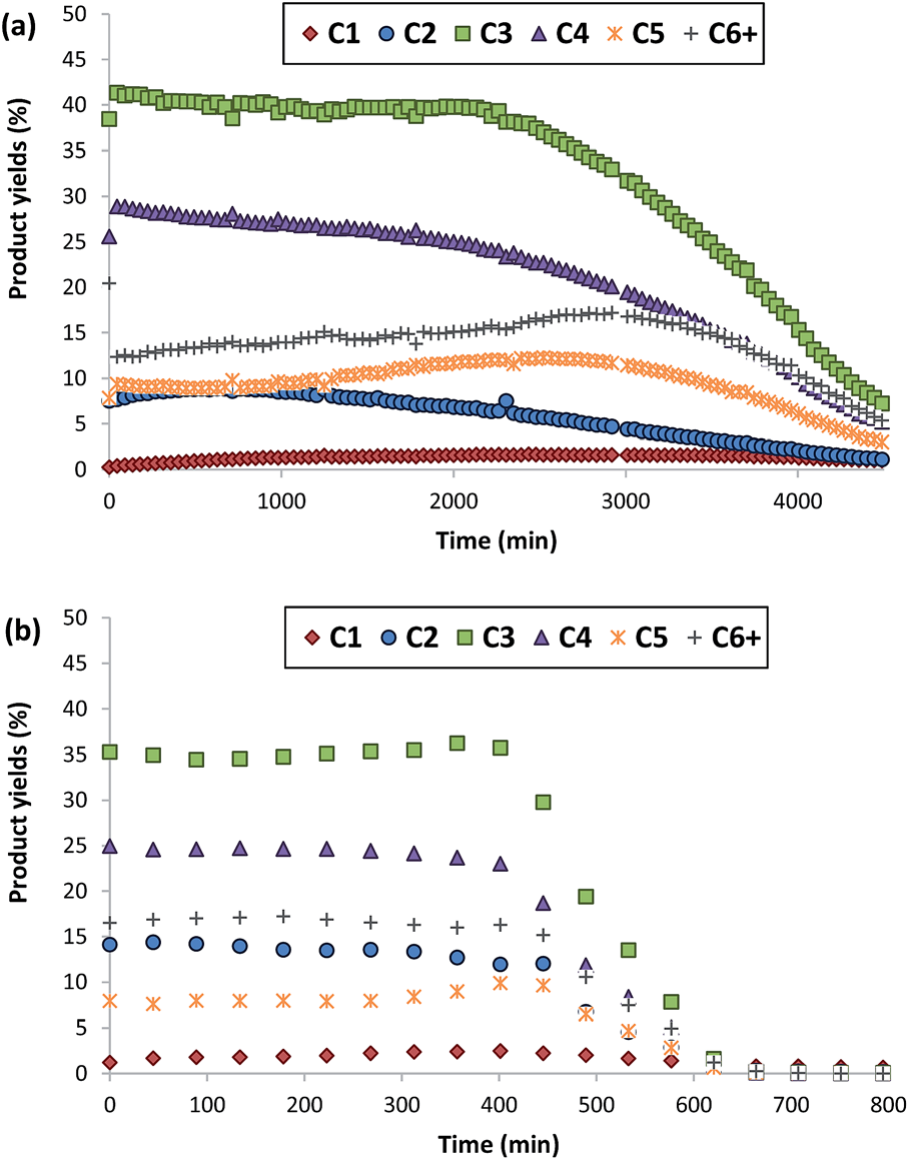
Product yields obtained using BMP-30 (a) and TPA-30 (b) as catalysts for the MTO reaction (conditions: *T* = 450 °C, WHSV = 10 h^–1^, *W*_cat_ = 50 mg).

**Fig. 10:**
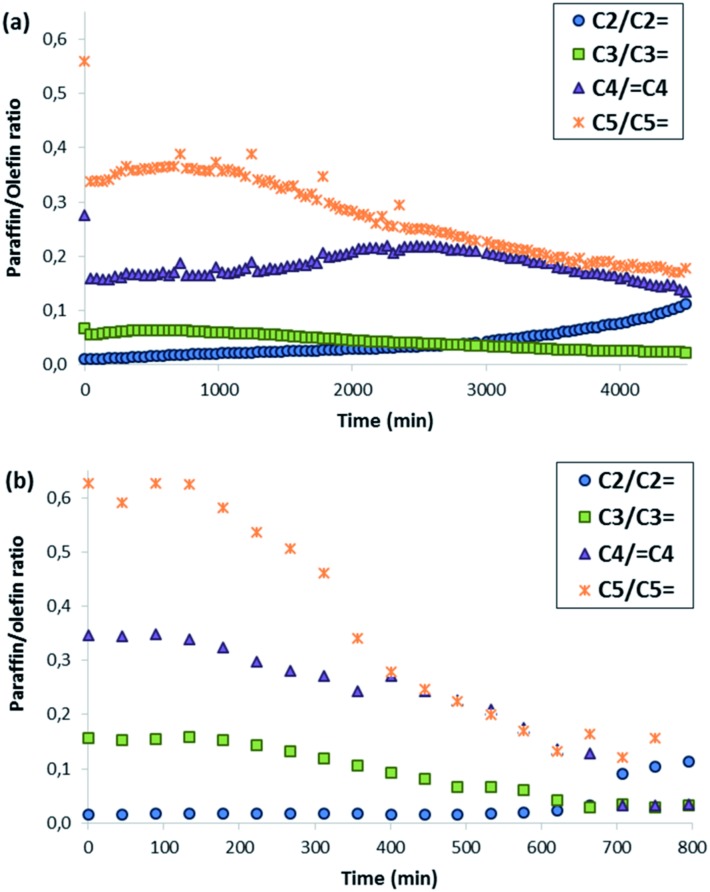
Hydrogen transfer ratios obtained using BMP-30 (a) and TPA-30 (b) as catalysts for the MTO reaction (conditions: *T* = 450 °C, WHSV = 10 h^–1^, *W*_cat_ = 50 mg).

Both ZSM-5 zeolites synthesized in this work, BMP-30 and TPA-30, have been tested for the MTO reaction at 450 °C with a WHSV = 10 h^–1^ (see Experimental section). As it can be seen in Fig. 8, the ZSM-5 zeolite synthesized with the OSDA-BMP organic molecule, BMP-30, shows a much larger catalyst lifetime than the ZSM-5 zeolite synthesized using the classical TPA molecule. Indeed, the nanosized BMP-30 catalyst shows a methanol conversion dropping below 50% after 3900 minutes on stream, while the traditional ZSM-5 zeolite achieves 50% methanol conversion after 500 minutes (see Fig. 8), indicating that the BMP-30 is almost 8 times more active than the traditional ZSM-5. Since both catalysts present similar Si/Al molar ratio and similar Brønsted acidity (see Table 2), the remarkably lower crystal size of the BMP-30 catalyst mainly explains its improved resistance against deactivation by coke formation. Regarding product selectivities, the nanosized BMP-30 catalyst provides higher selectivities towards C_3_–C_5_ products (C_3_: 39–41%, C_4_: 25–28%, C_5_: 9–12%, see Fig. 9a) than traditional ZSM-5 zeolite (C_3_: 35–37%, C_4_: 23–25%, C_5_: 7–9%, see Fig. 9b). In addition, the nanosized BMP-30 catalyst also gives much lower hydrogen transfer ratios (measured as paraffin/olefin ratios, see C_2_/C_2_=, C_3_/C_3_= and C_5_/C_5_= in Fig. 10a and b for the BMP-30 and TPA-30 catalysts, respectively), resulting in higher yields towards the desired light olefins, such as propylene, butylene and pentene.

#### Alkylation of benzene with propylene to obtain cumene

The selective synthesis of cumene from benzene and propylene is a very important petrochemical reaction, since cumene is the chemical precursor to obtain phenol and acetone.^35^ The industrial catalysts for the liquid phase alkylation of benzene with propylene are based on medium or large pore zeolites, such as MWW or beta zeolites.^3^,^35^ However, the design of these medium and large pore zeolites with shorter diffusion pathways, such as in their nanocrystalline form, is highly desired to reduce the catalyst deactivation by an excessive olefin oligomerization or formation of multi-alkylated products.^26^,^36^

From the different nanosized beta zeolites synthesized using the simple OSDAs shown in Fig. 1, the BDMC6-15 sample has been selected for a preliminary evaluation of its catalytic properties for the benzene alkylation reaction. This selection has been made based on the fact that the *N*-butyl-*N*,*N*-dimethylcyclohexanaminium (OSDA-BDMC6) molecule used in its preparation can be easily synthesized from inexpensive cyclohexylamine-derived molecules, suggesting that if the nanocrystalline beta zeolite prepared using the OSDA-BDMC6 molecule outperforms the catalytic properties of other related nanocrystalline beta zeolites, it could be considered as a very promising catalyst candidate for a potential industrial implementation. For comparison purposes, the nanosized beta zeolite synthesized using TEA as OSDA (TEA-15, see Table 1), will also be tested for the benzene alkylation reaction with propylene. It is important to note that the selected beta zeolites show very small crystal sizes in their nanosized forms, and comparable Si/Al molar ratios (see BDMC6 and TEA-15 in Table 1).

**Fig. 11:**
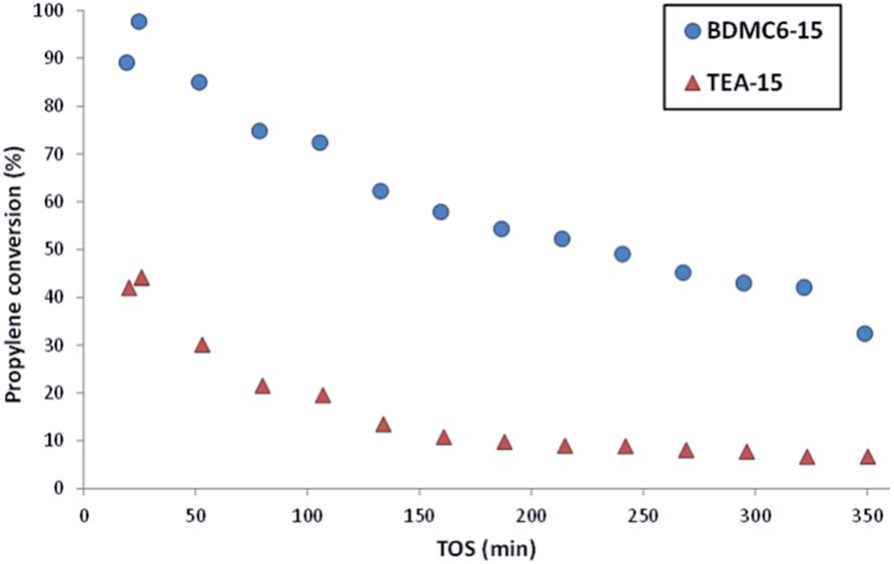
Propylene conversion with TOS for the liquid phase alkylation of benzene with propylene using nanosized betas as catalysts.

**Fig. 12:**
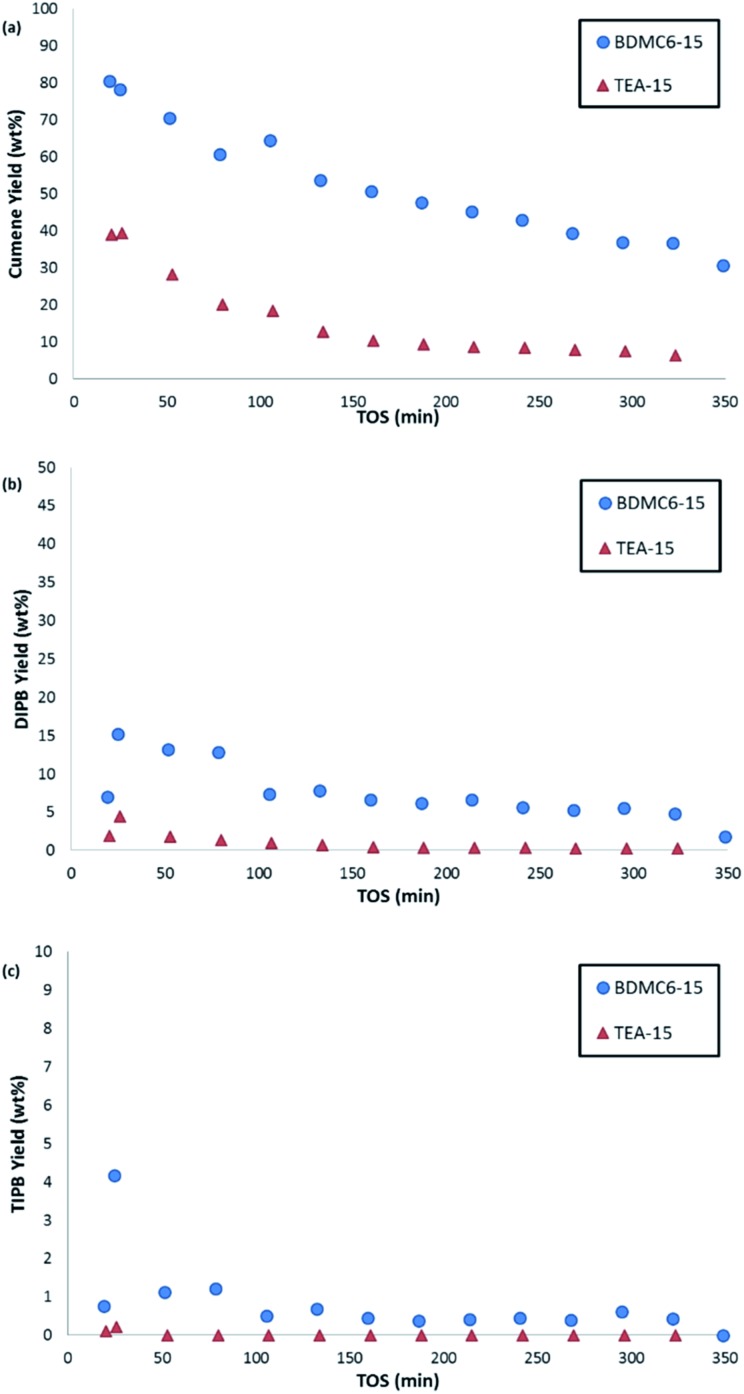
Selectivity to cumene (a), di-isopropylbenzene (b), and tri-isopropylbenzene (c) with TOS for the liquid phase alkylation of benzene with propylene using nanosized betas as catalysts.

The nanosized BDMC6-15 catalyst not only shows a remarkably higher initial propylene conversion and slower deactivation with time-on-stream than the nanosized TEA-15 beta zeolite (see Fig. 11), but also the yield towards the desired cumene product is considerably higher (see Fig. 12a). Di- and tri-isopropylbenzene complete the product distribution obtained with both catalysts (see Fig. 12b and c), with selectivity to non-alkylated products, and *n*-propylbenzene/cumene molar ratios below 1.0 wt% and 2 × 10^–3^, respectively. These better catalytic behavior observed with the BDMC6-15 catalyst could be mainly attributed to its crystal size, which would increase the molecular diffusion rates of reactants and products.

#### Oligomerization of olefins to liquid fuels

The oligomerization of light olefins is a very efficient procedure to obtain environmentally friendly synthetic fuels, such as gasoline and diesel, free of sulfur and aromatics.^37^ Along the years, different zeolite structures have been described as catalysts for the oligomerization of light olefins, observing, in general, that large diffusion pathways result in faster deactivation rates.^38^ This is particularly true for tridimensional large pore zeolites, where highly branched high molecular-weight products could be more easily formed.^39^

Here, two different nanosized beta zeolites will be tested as acid catalysts for the oligomerization reaction of 1-pentene. These nanosized beta catalysts are the same as those employed above for the benzene alkylation reaction, and the selected reaction conditions are 200 °C, 4.0 MPa, and two different space velocities (WHGS = 14.3 and 25 h^–1^, see Experimental section).

**Fig. 13:**
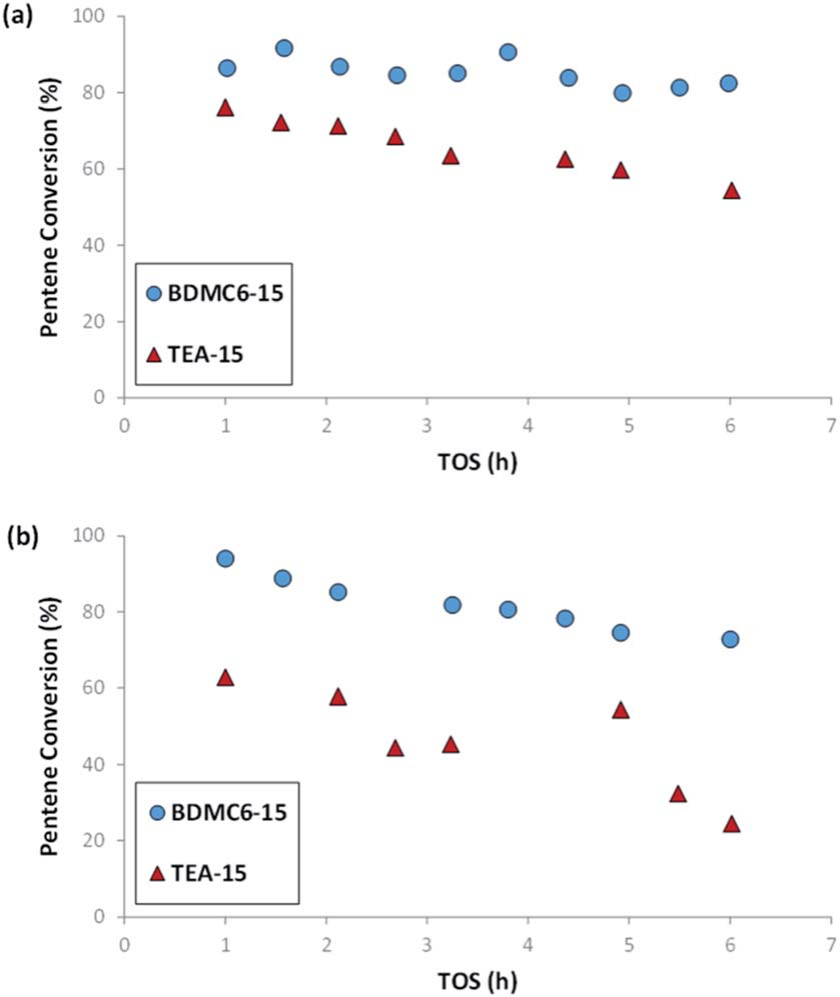
1-Pentene conversion *vs.* TOS for nanosized beta catalysts at WHSV = 14.3 (a), and 25 (b) h^–1^. *T* = 200 °C, *P* = 4.0 MPa, 60% mol olefin in the feed.

As shown in Fig. 13, the nanobeta zeolite presenting the lower crystal sizes, BDMC6-15, shows higher initial activity and stability towards deactivation with time-on-stream than the other nanobeta zeolite with slightly higher crystal size, which is the nanosized beta synthesized using TEA as OSDA. This trend is already observed when the tests are performed at a space velocity (WHSV) of 14.3 h^–1^, but the differences between BDMC6-15 and TEA-15 are much clearer when increasing WHSV to a value of 25 h^–1^ (see Fig. 13b).

**Fig. 14:**
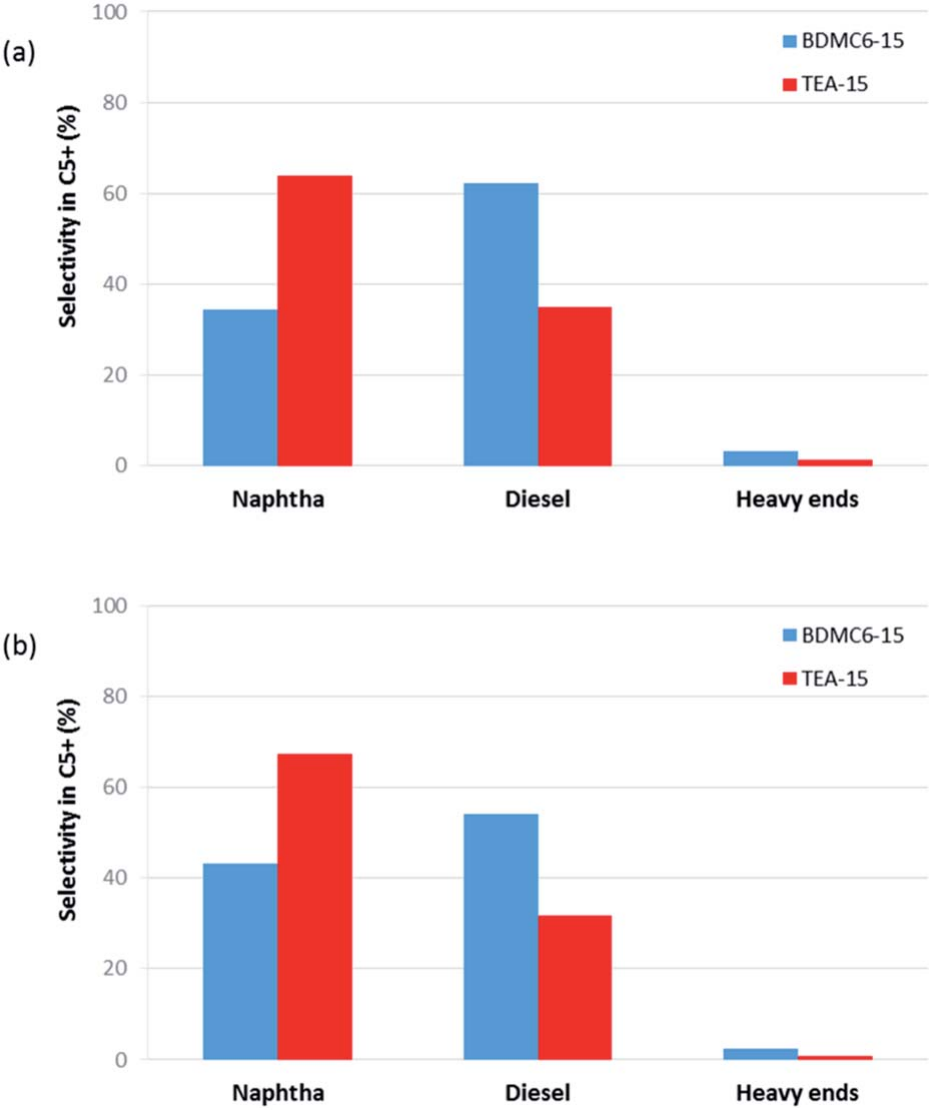
Selectivity within the accumulated C_5_^+^ liquid fractions for the nanosized beta catalysts at WHSV = 14.3 h^–1^, *T* = 200 °C, *P* = 4.0 MPa, 60% mol olefin in the feed. (a) TOS = 0–3 h, (b) TOS = 3–6 h.

**Fig. 15:**
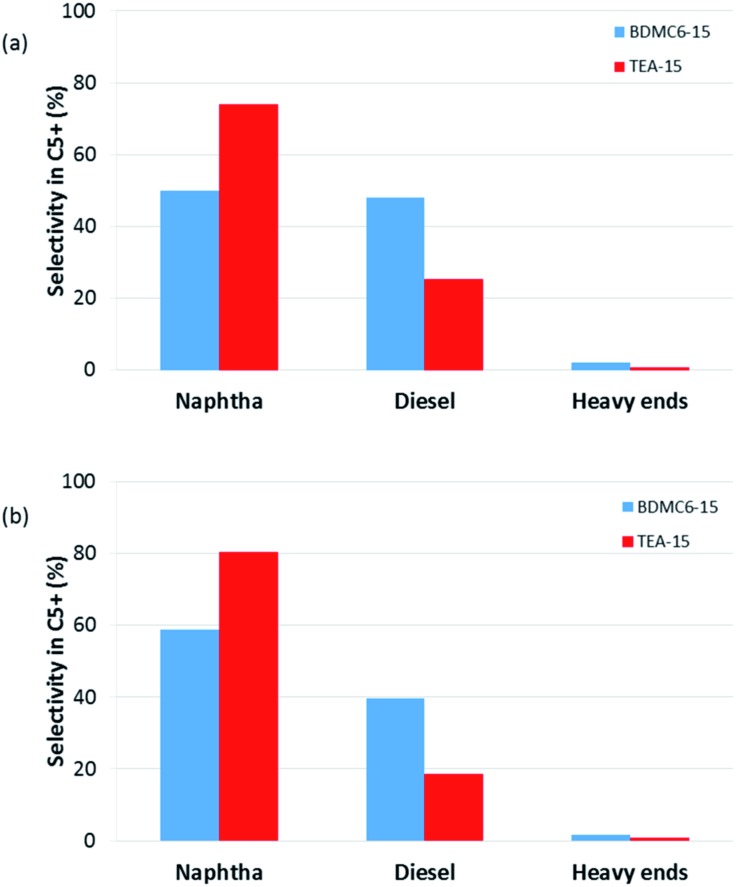
Selectivity within the accumulated C_5_^+^ liquid fractions for the nanosized beta catalysts at WHSV = 25 h^–1^, *T* = 200 °C, *P* = 4.0 MPa, 60% mol olefin in the feed. (a) TOS = 0–3 h, (b) TOS = 3–6 h.

Different product distribution could be observed depending on the physico-chemical properties of the nanobeta catalysts (see Fig. 14 and 15). When the nanobeta zeolites are compared at the lowest space velocity (WHSV = 14.3 h^–1^, see Fig. 14), the BDMC6-15 shows higher selectivity to diesel than the nanosized TEA-15 zeolites. This fact could be ascribed to the smaller crystal size of the BDMC6 nanobeta, which would favor the egression of the larger oligomers from zeolite crystals due to the reduced path lengths. When the space velocity is increased, the selectivity to diesel obtained with both catalysts is decreased, as could be expected due to the lower number of oligomerization steps.^40^ Still, even under these conditions of higher throughput, the BDMC6-15 beta is more selective to diesel than the beta zeolite synthesized in presence of TEA.

## 4. Conclusions

The efficient synthesis of the ZSM-5 and beta zeolites in their nanosized form has been described using simple dual-function-based monocationic organic molecules as OSDAs, presenting the combination of a short alkyl chain, *i.e.* butyl, and a cyclic ring within their structures. This methodology has been shown to be general for different OSDA molecules, including cyclic ammonium cations or cyclo-alkylated ammonium cations, where the ring size of the cycle can be varied. Depending on the ring size, nanosized ZSM-5 or beta zeolites have been obtained with average crystal sizes between 10–20 nm, excellent solid yields (above 90%), and controlled chemical compositions (Si/Al ∼ 15–30). ^27^Al MAS NMR spectroscopy reveals that most of the aluminum species remain in tetrahedral coordination within the crystalline frameworks of ZSM-5 and beta after calcination, resulting in nanosized materials with high Brønsted acidities, as measured by pyridine adsorption/desorption. The ZSM-5 and beta nanozeolites synthesized using simple alkyl-substituted cyclic ammonium cations as OSDAs have been tested in different industrially relevant reactions, such as MTO, benzene propylation or olefin oligomerization, presenting in all cases improved catalytic activity and product selectivity towards target products. These preliminary results allow envisioning that this synthesis methodology could be applied for the design of other nanocrystalline zeolites in a very efficient manner.

## Conflicts of interest

There are no conflicts to declare.

## References

[cit1] Davis M. E. (2014). Chem. Mater..

[cit2] Moliner M., Martínez C., Corma A. (2015). Angew. Chem., Int. Ed..

[cit3] Martínez C., Corma A. (2011). Coord. Chem. Rev..

[cit4] Cejka J., Centi G., Perez-Pariente J., Roth W. J. (2012). Catal. Today.

[cit5] Gallego E. M., Portilla M. T., Paris C., León-Escamilla A., Boronat M., Moliner M., Corma A. (2017). Science.

[cit6] Brandenberger S., Krocher O., Tissler A., Althoff R. (2008). Catal. Rev..

[cit7] Tosheva L., Valtchev V. P. (2005). Chem. Mater..

[cit8] Valtchev V., Tosheva L. (2013). Chem. Rev..

[cit9] Sarazen M. L., Doskocil E., Iglesia E. (2016). ACS Catal..

[cit10] Mintova S., Gilson J. P., Valtchev V. (2013). Nanoscale.

[cit11] Camblor M. A., Corma A., Valencia S. (1998). Microporous Mesoporous Mater..

[cit12] Mintova S., Valtchev V., Onfroy T., Marichal C., Knözinger H., Bein T. (2006). Microporous Mesoporous Mater..

[cit13] Bhoeman B. J., Babouchkina E., Mintova S., Valtchev V. P., Sterte J. (2001). J. Porous Mater..

[cit14] Van Grieken R., Sotelo J. L., Menendez J. M., Melero J. A. (2000). Microporous Mesoporous Mater..

[cit15] Iwakai K., Tago T., Konno H., Nakasaka Y., Masuda T. (2011). Microporous Mesoporous Mater..

[cit16] Bellussi G., Pollesel P. (2005). Stud. Surf. Sci. Catal..

[cit17] Yilmaz B., Muller U. (2009). Top. Catal..

[cit18] Zones S. I. (2011). Microporous Mesoporous Mater..

[cit19] Larlus O., Mintova S., Wilson S. T., Willis R. R., Abrevaya H., Bein T. (2011). Microporous Mesoporous Mater..

[cit20] Choi M., Na K., Ryoo R. (2009). Chem. Commun..

[cit21] Zhu J., Zhu Y., Zhu L., Rigutto M., van der Made A., Yang C., Pan S., Wang L., Zhu L., Jin Y., Sun Q., Wu Q., Meng X., Zhang D., Han Y., Li J., Chu J., Zheng A., Qiu S., Zheng X., Xiao F. S. (2014). J. Am. Chem. Soc..

[cit22] Choi M., Na K., Kim J., Sakamoto Y., Terasaki O., Ryoo R. (2009). Nature.

[cit23] Kim Y., Kim K., Ryoo R. (2017). Chem. Mater..

[cit24] Mintova S., Grand J., Valtchev V. (2016). C. R. Chim..

[cit25] Burton A. W., WO2014/099261, 2014, assigned to ExxonMobil.

[cit26] Martinez-Franco R., Paris C., Martinez-Armero M. E., Martinez C., Moliner M., Corma A. (2016). Chem. Sci..

[cit27] Chiappe C., Pomelli C. S., Rajamani S. (2011). J. Phys. Chem. B.

[cit28] Corma A., Martínez-Soria V., Schnoeveld E. (2000). J. Catal..

[cit29] Kokotailo G. T., Lawton S. L., Olson D. H., Meier W. M. (1978). Nature.

[cit30] Emeis C. A. (1993). J. Catal..

[cit31] Treacy M. M. J., Newsam J. M. (1988). Nature.

[cit32] Stocker M. (1999). Microporous Mesoporous Mater..

[cit33] Tian P., Wei Y., Ye M., Liu Z. (2015). ACS Catal..

[cit34] Martin N., Li Z., Martıinez-Triguero J., Yu J., Moliner M., Corma A. (2016). Chem. Commun..

[cit35] Vermeiren W., Gilson J. P. (2009). Top. Catal..

[cit36] Margarit V. J., Martínez-Armero M. E., Navarro M. T., Martínez C., Corma A. (2015). Angew. Chem., Int. Ed..

[cit37] Paretello S., Molinari M., Bellussi G., Perego C. (1999). Catal. Today.

[cit38] Corma A., Martinez C., Doskocil E. (2013). J. Catal..

[cit39] Pater J. P. G., Jacobs P. A., Martens J. A. (1998). J. Catal..

[cit40] Tabak S. A., Krambeck F. J., Garwood W. E. (1986). AIChE J..

